# Initial Cluster Analysis

**DOI:** 10.1089/cmb.2017.0050

**Published:** 2018-02-01

**Authors:** Stephen F. Altschul, Andrew F. Neuwald

**Affiliations:** ^1^ National Center for Biotechnology Information, National Library of Medicine, National Institutes of Health, Bethesda, Maryland.; ^2^ Department of Biochemistry and Molecular Biology, Institute for Genome Sciences, University of Maryland School of Medicine, Baltimore, Maryland.

**Keywords:** cluster analysis, Minimum Description Length principle, Jeffreys' priors

## Abstract

**We study a simple abstract problem motivated by a variety of applications in protein sequence analysis. Consider a string of 0s and 1s of length** ***L*, and containing** ***D*** **1s. If we believe that some or all of the 1s may be clustered near the start of the sequence, which subset is the most significantly so clustered, and how significant is this clustering? We approach this question using the minimum description length principle and illustrate its application by analyzing residues that distinguish translational initiation and elongation factor** **guanosine triphosphatases (GTPases) from other P-loop GTPases. Within a structure of yeast elongation factor 1**$\alpha$**, these residues form a significant cluster centered on a region implicated in guanine nucleotide exchange. Various biomedical questions may be cast as the abstract problem considered here.**

## 1. Introduction

In the study of proteins, a variety of methods derive, from analyzing a multiple alignment of related sequences or a corresponding phylogenetic tree, predictions that a specific set of residues within a particular protein are functionally important (Lichtarge et al., 1996; Mihalek et al., 2004; Fischer et al., 2008; Sankararaman and Sjölander, 2008; Janda et al., 2012; Neuwald and Altschul, 2016). When a three-dimensional structure is available for that protein, the residues so identified may appear to form one or more spatial clusters perhaps indicative of active sites or other functional modules. How can we identify the set of residues that best constitute such a cluster, and determine whether the cluster is statistically significant or can be explained completely by chance?

Pioneering work on this question was done by Karlin and Zhu (1996), who suggested that orders for the amino acid residues in a protein other than their arrangement along the protein's backbone could be studied. Specifically, given a three-dimensional structure, one could reorder protein residues by their distance from a specific starting residue or from a specific point in space; by their nearest distance to a progressively growing residue set; by their distance to the center of mass of such a set; or by any other well-defined criterion. Karlin and Zhu (1996) proposed seeking clusters of particular residue types within these reordered sequences. We use the first of these reordering methods here, and elsewhere describe several others of utility in various biological contexts (Neuwald, Aravind and Altschul, submitted).

Our general approach diverges from Karlin and Zhu's in three specific ways. First, we focus on clusters of residues that are “distinguished” by a computer analysis of multiple alignments, rather than on residues having specific physical–chemical properties such as charge (Karlin and Zhu, 1996; Zhu and Karlin, 1996). This has no formal effect on our approach, only a motivational one. Second, we seek the most surprising initial cluster along a sequence. In other words, we seek to define the best division of a sequence into an initial segment with a high density of distinguished residues, and a terminal segment with a low density. Finally, we assume we are given a fixed number of distinguished residues along a sequence, in contrast to Karlin's approach, which assumes a fixed independent probability for a residue being distinguished. In other words, we derive statistics based on sampling without as opposed to with replacement.

Below we use the Minimum Description Length (MDL) principle (Grünwald, 2007) to formulate and analyze our problem in simple abstract terms, and then illustrate its application to a specific protein sequence and structure.

## 2. Formalization Using the MDL Principle

Assume we are given a sequence of length *L* consisting of *D* 1s and $( L - D )$ 0s. We wish to compare the hypothesis *H_1_* that the 1s cluster near the start of the sequence with the null hypothesis *H*_0_ that the 1s and 0s occur randomly.

The MDL principle defines a theory $\theta$ as a probability distribution ${P_ \theta}$ over the space of all possible sets of data. The description length (in bits) of a data set *S* given a theory $\theta$ is then defined as ${ \rm{DL}} ( S \vert \theta ) =- \mathop { \log } \nolimits_{2{ \kern 1pt} } [ {P_ \theta } ( S ) ]$. (Throughout this article, we assume $\log  { \rm s}$ to be base 2; when natural logarithms are needed, we use the notation ln.) A model $\mathfrak{M}$ is a parameterized set of theories, and the description length of *S* given $\mathfrak{M}$ is defined as ${ \rm{DL}} ( S \vert \mathfrak{M} ) = \mathop { \min } \nolimits_{ \theta \in \mathfrak{M}} { \rm{DL}} ( S \vert \theta )$. The MDL principle asserts that given multiple models to explain *S*, one should prefer the model $\mathfrak{M}$ that minimizes ${ \rm{DL}} ( S \vert \mathfrak{M} ) + { \rm{COMP}} ( \mathfrak{M} )$. Here, the description length or complexity ${ \rm{COMP}} ( \mathfrak{M} )$ of a model $\mathfrak{M}$ is the log of the number of effectively independent theories $\mathfrak{M}$ contains. A central element of MDL theory is the formal definition of ${ \rm{COMP}} ( \mathfrak{M} )$, and its calculation for specific models.

### 2.1. The description length of *S* given *H*_0_, and the complexity of *H*_0_

For our purposes, we take *H*_0_ to be the model consisting of a single Bernoulli trial theory for generating *S*, with the probability of a 1 taken as $P = D / L$, and the probability of a 0 taken as $Q = 1 - P$. We then have

\begin{align*}
 { \rm { DL } } ( S \vert { H_0 } ) = - \log { P_ { { H_0 } } } ( S ) = - \log \left( { P^D } { Q^ { L - D } } \right) = D \log \frac { L }  { D } + ( L - D ) \log \frac { L }  { { L - D } } , \tag { 1 } 
\end{align*}

which is *L* times the entropy of the Bernoulli trial. Because *H*_0_ contains only one theory, its complexity is ${ \rm{COMP}} ( {H_0} ) = \log ( 1 ) = 0$. Note that, more formally, we could treat *P* as a parameter estimated from the data, in which case the complexity of the resulting single-parameter model would be approximately ${ \textstyle \frac { 1 }  { 2 } } \log ( \pi L / 2 )$ (Grünwald, 2007). However, because we will assume *D* to be fixed in our model *H*_1_ as well, where its indeterminacy would add a similar complexity, we may avoid the complication of treating *D* as variable when we wish simply to compare *H*_0_ to *H*_1_.

### 2.2. The description length of *S* given *H*_1_

The hypothesis *H*_1_ may be understood as a single-parameter model, whose parameter *x* describes the location of a cut at a discrete point from 1 to $L - 1$ along the sequence *S*, thereby dividing it into an initial segment *S_1_* of length *x*, and a terminal segment *S_2_* of length $y = L - x$. If *S_1_* contains *D_1_* 1s, and *S_2_* contains ${D_2} = ( D - {D_1} )$ 1s, assume at first that *S_1_* is generated by Bernoulli trials with maximum-likelihood probability ${P_1} = {D_1} / x$ for a 1, and *S_2_* is generated by Bernoulli trials with probability ${P_2} = {D_2} / y$ for a 1. Naively, given a particular fixed value for our parameter *x*, the probability for the data *S* would seem to be

\begin{align*}
{K_x} ( S ) = P_1^{{D_1}}{ \kern 1pt} { ( 1 - {P_1} ) ^{x - {D_1}}}{ \kern 1pt} P_2^{{D_2}}{ \kern 1pt} { ( 1 - {P_2} ) ^{y - {D_2}}}. \tag{2}
\end{align*}

However, this equation estimates *P_1_* (and therefore implicitly *P_2_*) by maximum-likelihood from the data *S*, so *K_x_* is not a probability distribution over the space of all possible data. To avoid adding a second parameter and attendant complexity to the model *H*_1_, we may define the probability *P_x_* for *S* as its normalized maximum likelihood (NML) (Grünwald, 2007). This is simply

\begin{align*}
{P_x} ( S ) = {K_x} ( S ) { \kern 1pt} / { \kern 1pt} Z , \tag{3}
\end{align*}

where *Z* is the sum of ${K_x} ( S )$ over all length-*L* sequences having *D* 1s. Note that using counting formulas, one may calculate *Z* using at most $D + 1$ terms, corresponding to *D*_1_ equal to 0 to *D*. The description length of the data *S* under *H*_1_ is then ${ \rm{DL}} ( S \vert {H_1} ) = - \log [ { \max \nolimits_x}{P_x} ( S ) ]$.

### 2.3. The complexity of *H*_1_

We still need to calculate the complexity of the model *H*_1_, which is the log of the effective number of independent theories it contains. Intuitively, the problem is that while *P_x_* is a probability distribution for any fixed *x*, we allow *x* to take on values from 1 to $L - 1$, each of which will yield a ${P_x} ( S )$. When we select the maximum, *P_X_*, it again becomes a likelihood, and we need to discount ${P_X} ( S )$ for the multiple trials implicit in the $L - 1$ possible choices for *x*. The Bonferroni correction, which simply multiplies ${P_X} ( S )$ by $L - 1$, is much too conservative, so we seek the number *I* of effectively independent values of *x*. For one-parameter models parameterized by a continuous *x*, this is given by

\begin{align*}
I \approx \frac { 1 }  { { \sqrt { 2 \pi } } } \int \sqrt { { J_x } } dx , \tag { 4 } 
\end{align*}

where *J_x_* is the Fisher information associated with parameter *x* (Grünwald, 2007). However, because our *x* is discrete, we will study a continuous analog to our problem to approximate *J_x_* and *I*. Specifically, we take *x* as continuous, with domain $( 0 , L )$, and the number of 1s observed in the initial and terminal segments to be Poisson distributed. The NML probability for observing *D*_1_ 1s in the initial segment [and therefore ${D_2} = ( D - {D_1} )$ 1s in the terminal segment] is then given by

\begin{align*}
f ( { D_1 } ) = \frac { 1 }  { Z } { \kern 1pt } { \frac { \lambda _1^ { { D_1 } } { \kern 1pt } { e^ { - { \lambda _1 } } } }  { { D_1 } ! } } { \kern 1pt } { \frac { \lambda _2^ { { D_2 } } { \kern 1pt } { e^ { - { \lambda _2 } } } }  { { D_2 } ! } } , \tag { 5 } 
\end{align*}

where ${ \lambda _1}$ and ${ \lambda _2}$ are the Poisson parameters associated, respectively, with the initial and terminal segments, and *Z* is a normalization constant. Our assumption is that there are different probability densities ${ \Lambda _1}$ and ${ \Lambda _2}$ for the occurrences of 1s in the initial and terminal segments, so that when we vary *x* we can write

\begin{align*}
{ \lambda _1} = { \Lambda _1}x \quad { \rm{and}} \quad { \lambda _2} = { \Lambda _2}y , \tag{6}
\end{align*}

for constant ${ \Lambda _1}$ and ${ \Lambda _2}$. Combining Eqs. (5) and (6), we can view $f ( {D_1} )$ as a function of *x*, and derive the Fisher information for the parameter *x* (Grünwald, 2007) from

\begin{align*}
 { J_X } = - { \rm { E } } \left[ { { \frac { { d^2 } }  { d { x^2 } } } \ln f ( { D_1 } ) \vert x = X } \right] , \tag { 7 } 
\end{align*}

where ${ \rm{E}} [. ]$ denotes the expected value. We begin by observing that

\begin{align*}
\ln f ( {D_1} ) = {D_1} \ln x - { \Lambda _1}x + {D_2} \ln y - { \Lambda _2}y + C , \tag{8}
\end{align*}

where *C* is a constant, so that

\begin{align*}
 { J_X } = { \rm { E } } \left[ { { \frac { { D_1 } }  { { x^2 } } } + { \frac { { D_2 } }  { { y^2 } } } \vert x = X } \right] = { \frac { { \rm { E } } [ { D_1 } \vert x = X ] }  { { X^2 } } } + { \frac { { \rm { E } } [ { D_2 } \vert x = X ] }  { { Y^2 } } } , \tag { 9 } 
\end{align*}

where $Y = L - X$. From Eq. (5), one obtains ${ \rm { E } } [ { D_1 } ] = \frac { { { e^ { - D } } } }  { Z } \sum \nolimits_ { i = 0 } ^D { \frac { { i^ { i + 1 } } { j^j } }  { i! { \kern 1pt } j! } }$ and ${ \rm { E } } [ { D_2 } ] = \frac { { { e^ { - D } } } }  { Z } \sum \nolimits_ { i = 0 } ^D { \frac { { i^i } { j^ { j + 1 } } }  { i! { \kern 1pt } j! } }$, where $j = D - i$ and ${0^0}$ is defined as 1 by continuity. A shortcut arises by observing that the expressions for ${ \rm{E}} [ {D_1} ]$ and ${ \rm{E}} [ {D_2} ]$ are equal. Therefore, since ${D_1} + {D_2} = D$, we must have ${ \rm{E}} [ {D_1} ] = { \rm{E}} [ {D_2} ] = D / 2$, which then yields

\begin{align*}
 { J_X } = \frac { D }  { 2 } \left( { \frac { 1 }  { { { X^2 } } } + \frac { 1 }  { { { Y^2 } } } } \right). \tag { 10 } 
\end{align*}

Our primary use for *J_X_* is to calculate the number *I* of effectively independent values of *x*. Substituting *J_x_* from Eq. (10) into Eq. (4), but returning to discrete *x* to obtain a sum, we get

\begin{align*}
I \approx \frac { 1 }  { 2 } \sqrt { \frac { D }  { \pi } } \mathop \sum \limits_ { x = 1 } ^ { L - 1 } { \kern 1pt } \sqrt { \frac { 1 }  { { { x^2 } } } + \frac { 1 }  { { { y^2 } } } } . \tag { 11 } 
\end{align*}

It is simple enough to calculate the sum. However, since the sum's terms approach $1 / x$ and $1 / y$ for small *x* and small *y*, respectively, for large *L* the sum itself should approach $2 { \kern 1pt} \ln ( L ) + C$, or alternatively $2{ \kern 1pt} \ln ( KL )$, for some constants *C* and *K*. Computation shows that even for quite small *L* the sum may be very closely approximated by $2{ \kern 1pt} \ln ( 1.024{ \kern 1pt} L )$, yielding

\begin{align*}
I \approx \sqrt { \frac { D }  { \pi } } { \kern 1pt } \ln ( 1.024 { \kern 1pt } \;L ). \tag { 12 } 
\end{align*}

In the above analysis, we have made no distinction between cuts for which the initial segment has a greater density of 1s than the terminal segment, and those for which it has a lesser density. For the applications we envision, however, the latter case has no ready biological interpretation, so we wish to limit *H*_1_ to the former case. Over the space of sequences, this discards half of all cuts *H*_1_ allows, so when we impose this restriction we can effectively write ${ \rm{COMP}} ( {H_1} ) = \log ( I / 2 )$.

### 2.4. Flattening Jeffreys' priors

One subtlety is that the approach we have taken above is essentially equivalent to a Bayesian approach, in which Jeffreys' priors are assigned to parameter *x* (Jeffreys, 1946; Grünwald, 2007). Jeffreys' priors are proportional to the square root of the Fisher information, or in our case to $\sqrt {1 / {x^2} + 1 / {y^2}}$. Inspection shows this function to be U-shaped, diverging as *x* or *y* approaches 0, and with a minimum at $x = L / 2$. However, there is no biological reason to expect the location of real cut-points to follow such a prior. Indeed, as we will illustrate below, the approach so far described has a tendency to trim or extend biologically significant clusters unduly. For most applications it would be much more appropriate to specify flat or uniform priors for *x* in place of Jeffreys'. We may attempt to do this by maximizing not ${P_x} ( S )$ of Eq. (3), but rather

\begin{align*}
 { R_x } ( S ) = { \frac { { P_x } ( S ) }  { \sqrt { 1 / { x^2 } + 1 / { y^2 } } } } . \tag { 13 } 
\end{align*}

This evidently favors intermediate values of *x* at the expense of extreme values and is a move toward rendering the density of independent theories flat in *x*. (Although this approach does not have rigorous theoretical support, we will show below, by random simulation, that it is reasonably effective.) Following through, we define the “generalized description length of the data” as

\begin{align*}
{ \rm{D}}{{ \rm{L}}^*} ( S \vert {H_1} ) = - \log [ { \kern 1pt} \mathop { \max } \limits_x \ {R_x} ( S ) { \kern 1pt} ]. \tag{14}
\end{align*}

When this is done, we conjecture that the corresponding formula for the “generalized number of independent theories” is given by

\begin{align*}
 { I^* } \approx \sqrt { \frac { D }  { \pi } } ( L - 1 ). \tag { 15 } 
\end{align*}

As before, because we restrict *H*_1_ to initial segments with a greater density of 1s than terminal segments, we get a generalized complexity ${ \rm{COMP}}^{*} ( {H_1} ) = \log ( {I^*} / 2 )$. We will refer to this somewhat heuristic approach as using “Flattened” as opposed to Jeffreys' priors, and explore its potential advantages below.

## 3. Random Simulation

**FIG. 1.**
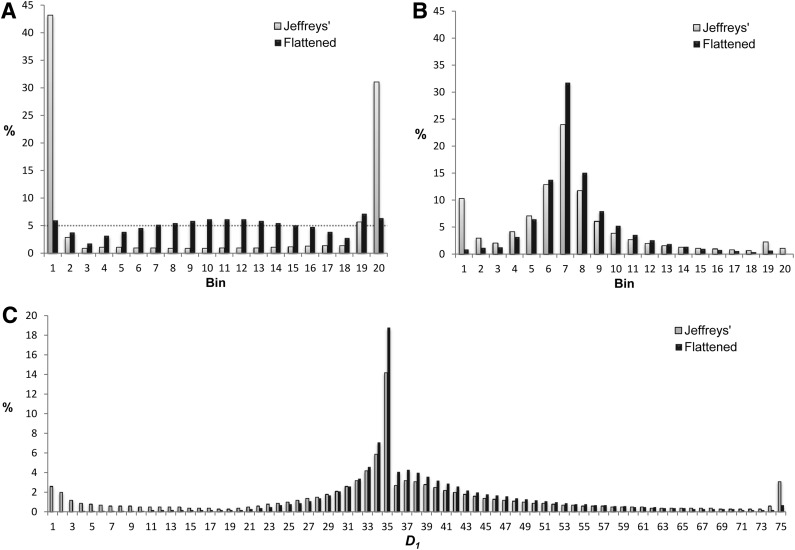
The optimization of *X* and *D*_1_ using Jeffreys' and Flattened priors. **(A)** Histogram for the optimal cut point *X* from ${10^6}$ random sequences with $L = 601$ and $D = 75$. Bins collect results for *X* from 1 to 30, 31 to 60, and so on. **(B)** Histogram for the optimal cut point *X* from ${10^6}$ random sequences with $L = 601$, 35 1s within the initial 200 positions, and 40 1s within the terminal 401 positions. **(C)** Histogram for the optimal *D*_1_ from the same experiment as for **(B)**.

The MDL principle says that we should prefer *H*_1_ to *H*_0_ when $${ \rm{DL}} ( S \vert {H_1} ) + { \rm{COMP}} ( {H_1} ) <$$$${ \rm{DL}} ( S \vert {H_0} ) +  { \rm{COMP}} ( {H_0} )$$. Treating each hypothesis as equally likely a priori, we may view the difference $$\Delta$$ between the two sides of this inequality as a log-odds ratio, and use the logistic function $$ { \frac { { e^ \Delta } }  { 1 + { e^ \Delta } } } $$ to convert this into a *p*-value; see p. 37 of Durbin et al. (1998). For any *S* for which *H*_1_ is preferred, there is a particular *X* that optimizes Eq. (3), and a corresponding number *D*_1_ of 1s in the initial segment. To elucidate the effect of Jeffreys' priors, we generated $${10^6}$$ random sequences of length $$L = 601$$ (thus allowing *X* to range from 1 to 600), each having $$D = 75$$ 1s, and recorded the optimal *X* for all sequences for which *H*_1_ was preferred to *H*_0_. We present in Panel A of Figure 1 a histogram (light bars) of the percentage of these *X*s falling into each of 20 bins (i.e., 1–30, 31–60, etc.). Due to the shape of Jeffreys' priors, bins with *X* near the extremes are strongly preferred. The asymmetry arises from our requirement that the initial segment have a greater density of 1s than the terminal. Applying Flattened rather than Jeffreys' priors to the identical sequences yields the alternative histogram (black bars). Although not flat, this histogram diverges, on average, much less from a uniform 5% in each bin (indicated by the dotted horizontal line) than does the Jeffreys' histogram.

In general, the choice of particular Bayesian priors becomes irrelevant for strong data, but can have a noticeable effect when data are weaker. For illustrative purposes, we consider artificially skewed sequences, also with $L = 601$ and $D = 75$, but with 35 1s placed randomly within an initial segment of length 200, and 40 1s placed randomly within a terminal segment of length 401. Generating ${10^6}$ such sequences, we used alternatively Jeffreys' and Flattened priors to find optimal *X*s and *D*_1_s; panels B and C of Figure 1 show the distributions of observed *X*s and *D*_1_s, respectively. Both Jeffreys' and Flattened priors are most likely to return an *X* within bin 7 (i.e., from 181 to 210) (Fig. 1B), which contains the “true” $X = 200$, as well as to return the “true” ${D_1} = 35$ (Fig. 1C). However, Jeffreys' priors have a marked tendency to trim or extend the “true” result. For 13.3% of sequences it returns an $X \le 60$ and for 3.4% an $X \ge 541$, compared to 2.1% and 0.7% for Flattened priors. Relatedly, for 7.5% of sequences it returns ${D_1} \le 5$ and for 4.6% ${D_1} \ge 71$, compared to 0.2% and 1.5% for Flattened priors. In addition, for this example Jeffreys' priors return, on average, somewhat less significant results: 62.5% of sequences with a *p*-value $\le 0.1$, as opposed to 86.4%, and 8.6% with a *p*-value $\le 0.01$, as opposed to 11.4%. For “true” cuts quite close to the sequence boundaries, however, Jeffreys' priors should have an advantage.

**FIG. 2.**
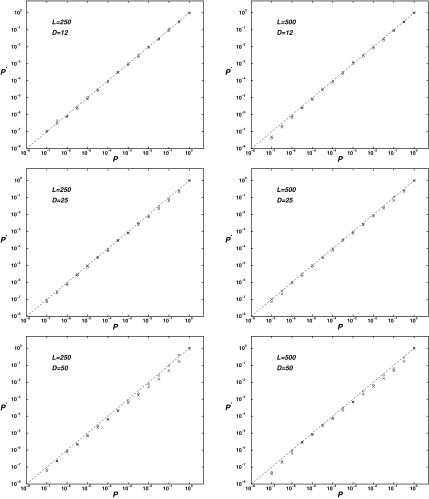
Observed *p* values ${P^*}$ as a function of calculated *p* values *P*. ${10^8}$ random sequences were generated for each of $L = 250$ and $L = 500$, and $D = 12$, $D = 25$, and $D = 50$. Jeffreys' and Flattened prior optimizations are represented by circles and crosses, respectively.

Given that Eq. (4) for *I* is an approximation, that we have made a further approximation in passing to a continuous analog of our problem to calculate *J_x_*, and that Eq. (15) for ${I^*}$ is conjectural, it is worth testing the accuracy of our analysis by random simulation. For $L = 250$ and $L = 500$, and for each of $D = 12$, $D = 25$, and $D = 50$, we generated ${10^8}$ random sequences, and maximized alternatively *P_x_* (Jeffreys' priors) and *R_x_* (Flattened priors). In Figure 2 we plot, for *P* from 1 to ${10^{ - 7}}$, the observed proportion ${P^*}$ of sequences with nominal *p*-value $\le P$. In these log–log graphs, theory is represented by the dotted lines with slope 1. The calculated *p* values tend to be somewhat conservative, that is, larger than the experimental ones. However, for $L = 500$, ${P^*}$ differs from *P*, within statistical error, by $\le 33 \%$ when $P \le 0.001$, and for $L = 250$ by $\le 39 \%$.

The conservative nature of our calculated *p*-values can be partially understood by noticing that the Poisson model we use to derive the Fisher information, and thus to calculate *I*, allows an arbitrary number of the *D* 1s to occur before any cut, whereas in fact at most *X* 1s may occur before the cut *X*, and symmetrically at most *Y* 1s may occur after such a cut. This aspect of our approximation should on average yield a greater error the larger *D* is with respect to *L*, as we observe in the examples here, as well as in others not shown. A simple heuristic correction that renders the calculated *P* on average in closer agreement with ${P^*}$ is to multiply either *I* or ${I^*}$ of Eqs. (4) and (15) by $1 - r$, where $r \approx D / L$ is the proportion of $( {D_1} , X )$ pairs allowed by the Poisson model but excluded by the discrete considerations that ${D_1} \le X$ and ${D_2} \le Y$. The resulting corrected values of *P* remain on average conservative but, in all cases considered, now differ from experiment by $\le  28 \%$ when $P \le 0.001$. We use this correction when calculating *p* values in the following section.

## 4. Application

To apply our theory to a particular protein we require, (a) a criterion for “distinguishing” particular amino acid residues, which we then represent as 1s, with all others represented as 0s, and (b) a criterion for ordering these residues, which in general does not correspond to the order imposed by the protein's backbone. Typically we distinguish residues based on a prediction of their importance or relevance, which may arise from the analysis of a multiple alignment, and we order residues based on some definition of structural distance, possibly from a fixed point in space. We may allow a particular, selected residue to provide such a point, in which case this “index” residue is omitted from the sequence.

As an illustration, we here analyze the P-loop GTPase domain (Hall, 2000) of the elongation factor eEF1A. GTPases constitute a large, functionally diverse protein superfamily (Leipe et al., 2002), for which hundreds of thousands of sequences are currently available. We aligned 127,418 GTPase domain sequences that were $< 95 \%$ identical and partitioned these sequences into subgroups, based on the particular patterns of residues characteristic of each subgroup (Neuwald, 2011, 2014). eEF1A was assigned to a set of translation initiation and elongation factors (TIEFs), and we used this subgroup's pattern of characteristic residues to distinguish $D = 20$ of eEF1A's $L = 158$ residues. Next, we used the structure of yeast eEF1A bound to the guanine nucleotide exchange factor (GEF) eEF1B$\alpha$ (pdb: 1g7c) (Andersen et al., 2001) to order eEF1A's residues, as follows. GEFs catalyze the exchange of guanosine diphosphate (GDP) bound to the GTPase domain, a process for which disruption of the GTPase ${ \rm{M}}{{ \rm{g}}^{ +  + }}$ binding site is believed to play a critical role. A lysine residue (K205) in eEF1B$\alpha$ appears to play a critical role in nucleotide exchange: K205 inserts into the ${ \rm{M}}{{ \rm{g}}^{ +  + }}$ binding site of eEF1A and is lethal when mutated. For this reason, we ordered eEF1A's residues by their distance from K205 of eEF1B$\alpha$.

Applying our approach with Flattened priors to the resulting sequence of 0s and 1s yields a cut at $X = 88$ with ${D_1} = 20$, that is, with all 20 distinguished residues in the initial segment; this division is highly significant, with *p*-value $5.4 \times {10^{ - 6}}$. If we distinguish instead the $D = 20$ residue positions that are most distinctive of all P-loop GTPases, we find an optimal cut with $X = 66$ and ${D_1} = 17$, and *p*-value $1.1 \times {10^{ - 6}}$. Jeffreys' priors yield the same cuts in both cases, although with different but still highly significant *p* values.

**FIG. 3.**
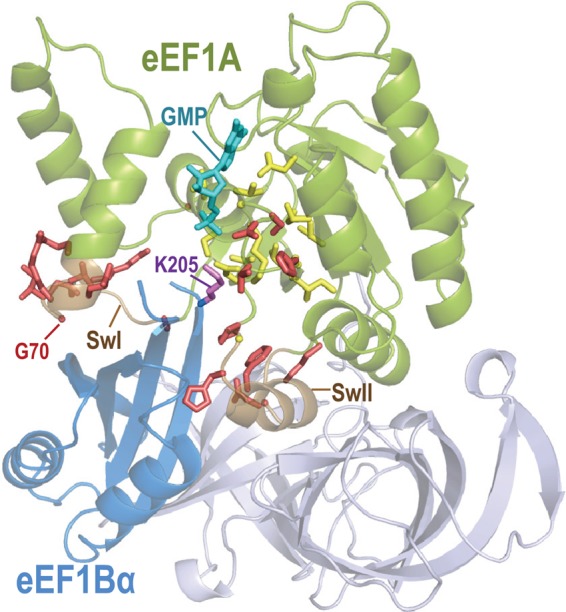
Initial cluster analysis of residues within the yeast elongation factor eEF1A GTPase domain bound to the nucleotide exchange factor eEF1B$\alpha$ (pdb_id: 1g7c). Color scheme: eEF1A GTPase domain, *green*; eEF1A switch I and II regions, *brown*; eEF1A domains II and III, *gray*; eEF1B$\alpha$, *marine blue*; GMP, *cyan*; side chains of TIEF-specific and GTPase-conserved residues included in initial clusters, *red and yellow*, respectively (two side chains common to both clusters are colored *red*). K205 of eEF1B$\alpha$ and G70 of eEF1A, which were used as (alternative) focal points, are indicated. GMP, guanosine-5′-monophosphate; TIEF, translation initiation and elongation factor.

In Figure 3, we show these clusters within the eEF1A-eEF1B$\alpha$ crystal structure. The 20 TIEF-specific residues and 17 GTPase-specific residues surround the bound eEF1B$\alpha$ and map to the P-loop and switch I and II regions, which undergo functionally relevant conformational changes (Hall, 2000) associated with both sensing GTP versus GDP and with nucleotide exchange. Both K205 and the bound ${ \rm{M}}{{ \rm{g}}^{ +  + }}$ ion are positioned near both the active site (generally conserved in all GTPases) and the switch I and II regions, which sense and transmit to other cellular components whether the domain is bound to GDP or to GTP. These facts suggest why residue sets characteristic for TIEF and for GTPase sequences should cluster near K205 of eEF1B$\alpha$.

**FIG. 4.**
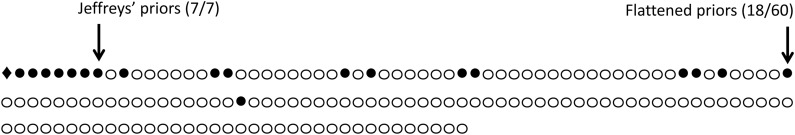
Cut points obtained using Jeffreys' priors versus Flattened priors. The same TIEF-specific analysis was performed as for Figure 3, except that G70 of eEF1A was used as a focal point instead of K205 of eEF1B$\alpha$. The *black diamond* represents the index residue G70; *black dots* and *open circles* represent 1s and 0s (i.e., discriminating and nondiscriminating residues), respectively. The fraction of discriminating residues in each initial cluster is shown parenthetically.

To illustrate how Flattened and Jeffreys' priors can yield different results, we performed the same TIEF-specific analysis, except with residues ordered by their distance from G70 of eEF1A rather than from K205 of eEF1B$\alpha$, shifting the focus of analysis to the switch I region. (The index residue G70 is accordingly ignored, yielding $D = 19$ and $L = 157$.) Jeffreys' priors return a cut with $X = 7$, ${D_1} = 7$, and $p = 2.4 \times {10^{ - 7}}$, whereas Flattened priors return a cut with $X = 60$, ${D_1} = 18$, and $p = 3.3 \times {10^{ - 7}}$; see Figure 4. In this case, Jeffreys' priors favor a cut near the start of the sequence, but Flattened prior eliminates this bias and, notably, identifies nearly the same cluster as before. Both cuts and their associated clusters have biologically relevant interpretations, although with somewhat different focuses.

## 5. Conclusion

Given a string of 0s and 1s, we have developed two methods to divide it into initial and terminal segments, with high and low concentrations of 1s, respectively, and to assess whether these segments differ significantly in their concentrations of 1s. The first method finds the maximum-likelihood division, but has a tendency to cut the sequence near its ends, which arises from the implicit use of Jeffreys' priors for the cut location. The second method seeks to counteract this tendency by flattening the implicit priors. C code implementing these methods is available from the authors on request. We have illustrated these methods by using them to identify statistically significant spatial clusters among residues that distinguish translational initiation and elongation factor GTPases from other P-loop GTPases. We will describe extensive applications elsewhere (Neuwald, Aravind and Altschul, submitted). Potential applications of our approach extend beyond protein sequence/structural analysis. One such application, for instance, would be to look for a significant association between a particular human microbiome-associated disease and RNA-Seq expression levels of a candidate microbial gene postulated to be responsible for the disease symptoms. In this case, both symptomatic and asymptomatic subjects could be ordered from highest to lowest levels of microbial gene expression; significant clustering of symptomatic subjects near the start point would indicate an association. Additional biomedical applications may similarly be devised.
